# Study on the mechanism of puerarin against osteoarthritis from ferroptosis based on network pharmacology and bioinformatics

**DOI:** 10.1007/s00210-023-02653-9

**Published:** 2023-08-07

**Authors:** Wenxiang Deng, Wenan Zhang, Qinghu He

**Affiliations:** 1 College of Integrated Traditional Chinese and Western Medicine, Hunan University of Chinese Medicine, Changsha, 410208 Hunan China; 2 Department of Rehabilitation and Healthcare, Hunan University of Medicine, Huaihua, 418000 Hunan China

**Keywords:** Network pharmacology, Ferroptosis, Puerarin, Osteoarthritis, Inflammatory factor

## Abstract

Network pharmacology and bioinformatics were used to study puerarin’s molecular mechanism in treating osteoarthritis from the perspective of ferroptosis, revealing a new treatment target. Ferroptosis-related targets were obtained from FerrDb. Puerarin action targets were retrieved from TCMSP, Pharmmappe, SwissTargetPrediction, and Targetnet databases, and supplemented with PubMed. The gene expression profiles of GSE12021, GSE55235, and GSE82107 were obtained using “Osteoarthritis” as the search term in the GEO database, and the differential expression gene screening analysis was performed for osteoarthritis. The intersection targets between puerarin, iron death, and osteoarthritis were obtained using Venn diagrams. GO and KEGG analyses were conducted with R software. Molecular docking and visualization of puerarin and core targets were performed using Autodock Vina and PyMol software. The effects of puerarin on the cell viability and the TNFα, IL6, and Ilβ levels of human inflammation articular chondrocytes were tested in vitro experiments. Puerarin, ferroptosis, and osteoarthritis share four targets: PLIN2, PTGS2, VEGFA, and IL6. GO enrichment analysis showed that puerarin maintained the blood-brain barrier, regulated peptide serine phosphorylation, and had anti-inflammatory effects. KEGG analysis showed that puerarin’s anti-inflammatory effects were mainly through VEGF, IL-17, C-type lectin receptor, HIF-1, TNF, and other signaling pathways. Puerarin closely bound PLIN2, PTGS2, VEGFA, and IL6 targets in molecular docking. In vitro, puerarin prevented osteoarthritis. Network pharmacology and bioinformatics explained puerarin’s multi-target and multi-pathway treatment of OA, which may be related to ferroptosis, and confirmed its anti-inflammatory effect.

Osteoarthritis (OA), a known degenerative disease, is a chronic joint disease that mainly leads to degenerative changes in articular cartilage and secondary osteoporosis, which is characterized by fibrosis, chapping, ulceration, and wear of articular cartilage due to a variety of factors (Abramoff and Caldera 2020). This disease belongs to traditional Chinese medicine (TCM) “Bi syndrome” category. Pro-inflammatory cytokines such as IL-1β, TNF-α, and IL-6, which are key compounds in the pathogenesis of osteoarthritis, may lead to the loss of articular cartilage homeostasis through metabolic changes and significantly accelerate joint injury (Wang and He 2018).

Ferroptosis is an iron-dependent oxidative cell death (Dixon et al. 2012). Ferroptosis and iron have important effects on the occurrence of OA, and iron death may act on osteoarthritis through oxidative stress, REDOX, and inflammation induction (Zhe et al. 2023). Ferroptosis can also trigger innate immunity, release inflammatory mediators, and activate inflammatory responses in the body (Mao et al. 2020). Changes in the typical physiological and metabolic properties of synovium produce inflammatory mediators such as IL-1β, TNF-α, and IL-6, which increase the uptake of transferrin and non-transferrin binding iron by monocytes and by synovium fibroblasts (Telfer and Brock 2004).

Puerarin, an isoflavone compound extracted from Pueraria lobata root, has been shown to have a variety of bioactive functions such as anti-inflammatory, anti-oxidation, and anti-virus (Zhou et al. 2014). Puerarin has a significant effect on the treatment of inflammatory diseases, such as cardiovascular disease (Li and Chen nd), diabetes (Liang et al. 2022), Alzheimer’s disease (Liu et al. 2019), cancer (Duan et al. 2021), and osteoarthritis (Peng et al. 2019). Therefore, puerarin treatment of osteoarthritis may be related to ferroptosis, but the relevant mechanism of puerarin regulating ferroptosis against osteoarthritis is still unclear.

Network pharmacology is a new method combining computer science and medicine (Hopkins 2008), and its interaction network based on “multiple genes,” “multiple targets,” and “multiple channels” is consistent with the thinking mode of TCM.

In this study, network pharmacology was used to explore the common genes of puerarin, ferroptosis, and osteoarthritis, and to predict the target and mechanism of puerarin regulating ferroptosis to inhibit osteoarthritis, to verify the effect of puerarin on the expression of inflammatory factors related to ferroptosis of osteoarthritis in vitro, and to provide ideas for the development and clinical application of anti-osteoarthritis drugs.

## Materials and methods

### Puerarin targets

The target of puerarin was searched in TCMSP (https://old.tcmsp-e.com/tcmsp.php/) using “puerarin” as the key word. Canonical SMILES and chemical structural formulae of puerarin were obtained in Pubchem (https://pubchem.ncbi.nlm.nih.gov/).

The targets of puerarin were searched through Pharmmappe (http://www.lilab-ecust.cn/pharmmapper/), SwissTargetPrediction (http://www.swisstargetprediction.ch/), and Targetnet databases (http://targetnet.scbdd.com/). The targets of puerarin were screened according to the principle of probability >0 in SwissTargetPrediction database and Prob>0 in Targetnet database, and the targets were supplemented in combination with literature in PubMed database. After merging, duplicate data were removed, and UniPort protein standardization database (https://www.uniprot.org/) was used to uniformly transform puerarin related targets.

### OA targets

The GSE12021, GSE55235, and GSE82107 datasets were downloaded from the GEO database (https://www.ncbi.nlm.nih.gov/geo/), which included 30 OA groups and 26 control groups. Batch effect is eliminated through the “SVA” package of R software. The limma package of R software was used for data analysis, the absolute value of logFC was set to be > 1, and *p* value was < 0.05 after correction. Genes with differential expression in the chip data were screened out, and visualized in the form of volcano map and heat map.

### Common genes of puerarin, ferroptosis, and osteoarthritis

Genes related to ferroptosis, such as “Driver,” “Suppressor,” and “Marker,” were retrieved from FerrDb database (http://www.zhounan.org/ferrdb/). The acquired puerarin target genes, differentially expressed genes, and ferroptosis related genes were interposed, and Veen was used to make the intersection and draw Venn diagram. The intersection genes were the common genes of puerarin, ferroptosis, and osteoarthritis.

### GO enrichment analysis and KEGG enrichment analysis

The GO and KEGG enrichment analysis of intersection genes was performed by the “clusterProfiler” package of R software, and the significant enrichment results were visualized by the ggplot2 package.

### Molecular docking

The common genes obtained in “1.3” were imported into the Uniprot database to obtain gene numbers, and the numbers were imported into the RCSB PDB database to obtain the protein structures encoded by the genes, and the receptors were selected and downloaded in PDB format.

Pymol software (input instruction: remove solvent; remove organic) was then used to remove water molecules and small molecules. The 3D structure of puerarin was obtained from the PubChem database and saved in SDF format. The spatial structure was optimized by Chemoffice software and saved in PDB format. The common genes of puerarin, ferroptosis, and OA were hydrogenated and docking boxes were created by AutoDock 1.5.6, and the docking of these genes with puerarin was performed by vina.

### In vitro experiments

#### Cell lines

Human articular chondrocytes (batch number REXFRHPZWA) were purchased from Procell Life Science &Technology Co., Ltd.

#### Drugs and reagents

Puerarin (batch number 27535) and celecoxib (batch number 19552) were purchased from MedChemexpress Biotechnology Inc. IL-1β (batch number 0606B95-1) and sterile ultrapure water (batch number A20353) were purchased from Multisciences (Lianke) Biotech, Co., Ltd. Human articular chondrocytes complete medium (batch number WH01112208SP08), 0.25% trypsin solution (batch number WH1622G051), and PBS buffer (batch number WH0022A071) were purchased from Procell Life Science &Technology Co., Ltd. CCK8 proliferation cell viability assay kit (batch number KL0306TV0989) was purchased from Elabscience Biotechnology Co., Ltd. Human IL-1β, IL-6, and TNF-α ELISA kit (batch number 10/2022) were purchased from Shanghai Enzyme-linked Biotechnology Co., Ltd.

#### Instrument

MB-530 multifunctional microplate analyzer (Shenzhen Huisong Technology Development Co., LTD.), Heal Force HF151-CO^2^ carbon dioxide incubator (Shanghai Ding International Trading Co., LTD.)

### Experimental verification that puerarin has anti-osteoarthritis effect

#### Culture and passage of articular chondrocytes

Human articular chondrocytes were first cultured at 37 ℃ with 5% CO^2^. The cells in logarithmic phase were cultured; when the cells reached 70–80% confluence, the articular chondrocytes were treated with complete culture medium containing IL-1β (10 ng/mL) for 24 h. Human articular chondrocytes were cultured in 5% CO^2^, 20% O^2^, 37 ℃ incubator for 2 days, and then changed to the medium. The cells were passaged after the fusion degree reached 70–80%. When the fusion degree of the second-generation chondrocytes reached 70–80%, the supernatant was discarded, and 1 mL of 0.25% trypsin was added, rinsed with PBS, and centrifuged at 1000 r/min for 5min. After mixing, the cells were divided into 25-cm^2^ cell culture flasks, placed into 5% CO^2^ incubator at 37 ℃ for routine culture and passage, and observed regularly under an inverted microscope.

#### Cell activity was detected by CCK-8 assay

The effects of different concentrations of puerarin on the proliferation of articular chondrocytes were detected by CCK-8 kit. A total of 5×103 Wells were seeded in 96-well plates and divided into blank control group, different concentrations of puerarin group (5 μM, 25 μM, 50 μM, 100 μM, 200 μM) and celecoxib group (5 μM, 10 μM, 20 μM, 100 μM, 200 μM), with 6 multiple Wells in each group. After 24 h of cell adherence, the cells were replaced with new medium and the non-adherent cells were discarded, and then the cells were cultured for 24 h and 48 h. Then, 10 μL CCK-8 solution was added to each well, gently shaken and mixed, and then routinely cultured for 2 h. The optical density value at 450-nm wavelength was detected by microplate reader to determine the proliferation of articular chondrocytes with different concentrations of puerarin and celecoxib.

#### Experimental grouping

The second generation of articular chondrocytes were divided into blank control group, model group (10 ng/mL IL-1β), puerarin group (10 ng/mL IL-1β+10uM puerarin), and celecoxib group (10 ng/mL IL-1β+10uM celecoxib) according to the different cultures. After 24 h of intervention, 10 multiple holes were set in each group.

#### ELISA was used to detect the related inflammatory factors

The operation was carried out according to the instructions of the ELISA kit, cell culture medium was added to the blank well, and the test samples were added to the sample well. Each group had 10 repeated detection samples, and the 450-nm wavelength was detected by microplate reader.

### Statistical analysis

SPSS 22.0 software was used for processing, measurement data in line with normal distribution were expressed as (x±s), t test was used for comparison between two groups, one-way analysis of variance was used for comparison between multiple groups, and LSD-t test was used for pairwise comparison between groups. *p* < 0.05 was considered statistically significant.

## Results

### Targets of puerarin

**Fig. 1:**
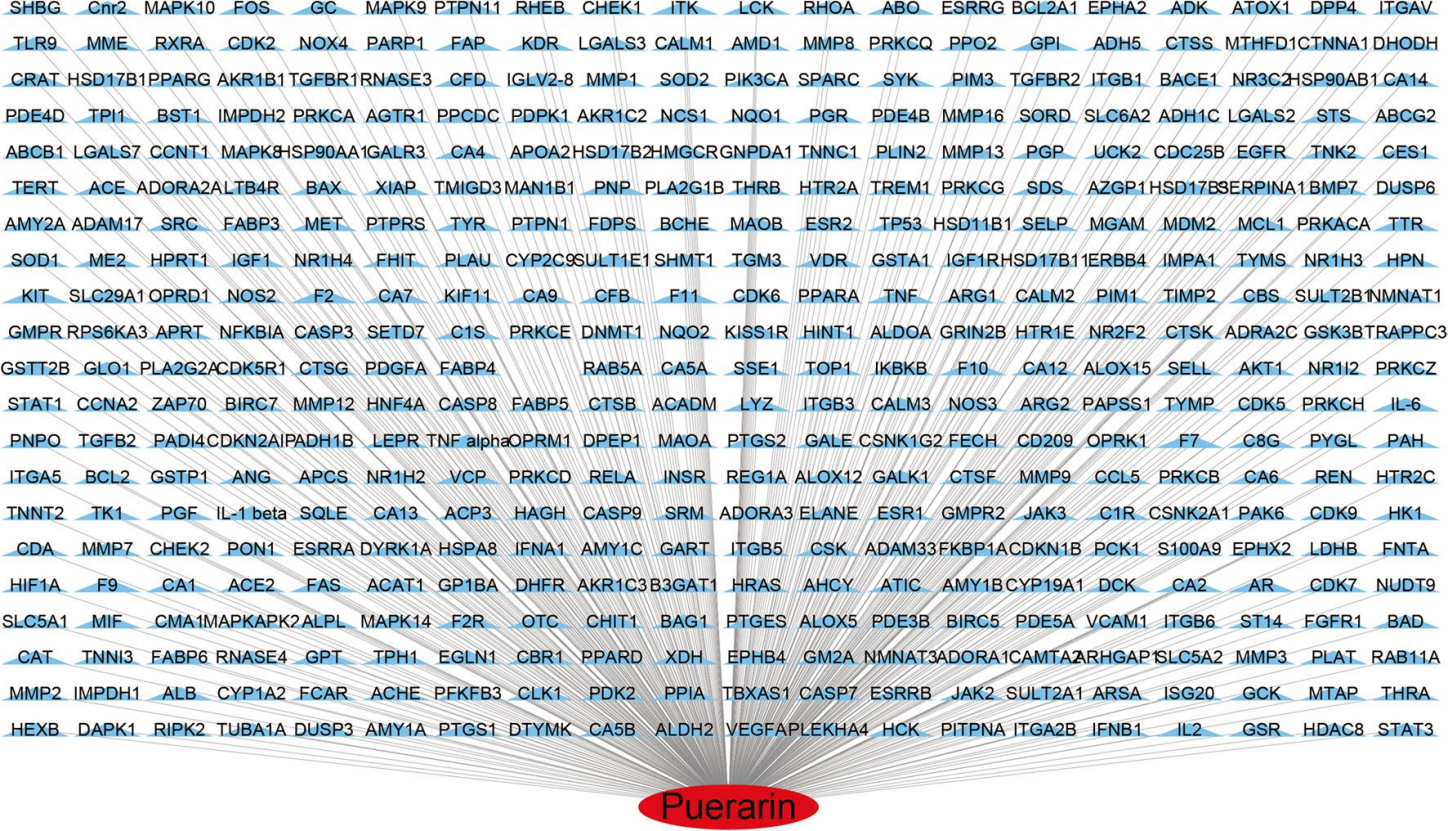
Puerarin-related targets

The potential targets related to puerarin were searched through TCMSP, Pharmmappe, SwissTargetPrediction, Targetnet database and literature, and a total of 419 targets were obtained after screening and reselection. Based on Cytoscape 3.7.1 software, a network diagram of puerarin targets was obtained, where red represents puerarin and blue represents the targets of puerarin action, as shown in Fig. 1.

### Differentially expressed genes in OA

**Fig. 2:**
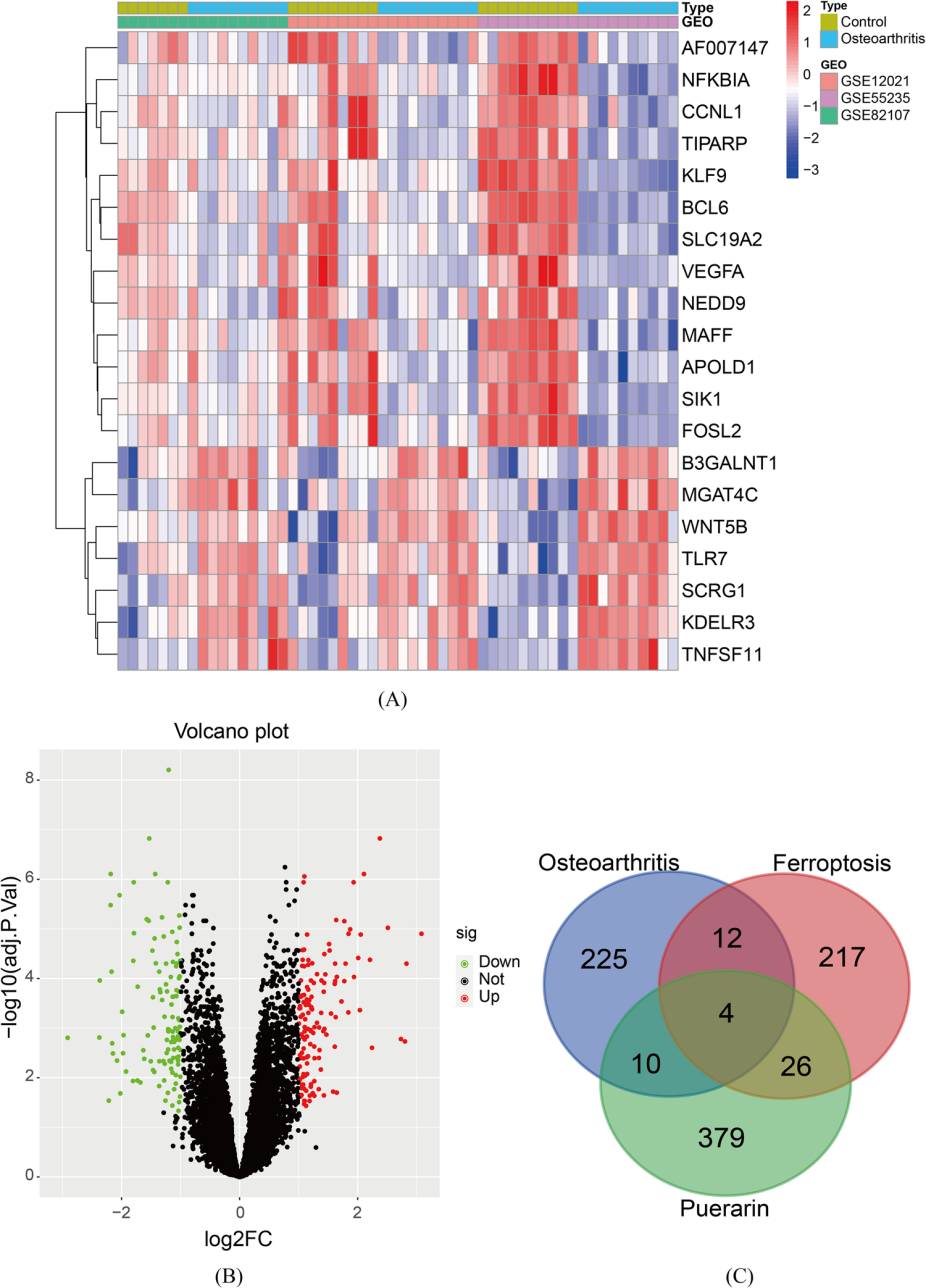
Heat map and volcano map of differential genes of OA and Venn diagram of puerarin, OA and ferroptosis. **A** Heat map of differential genes in OA. **B** Volcano map of differential genes in OA. **C** Venn map of common genes for puerarin, OA, and ferroptosis

After analyzing the data of chip GSE12021, GSE55235, and GSE82107, 251 genes with significant effects on the occurrence and development of OA were obtained, including 135 upregulated genes and 116 downregulated genes, as detailed in Fig. 2A and B.

### Common genes of puerarin, ferroptosis, and osteoarthritis

A total of 259 ferroptosis-related genes were obtained from FerrDb database, and 4 common genes of puerarin, ferroptosis, and osteoarthritis were obtained by overlapping the genes obtained in “Targets of puerarin” and “Differentially expressed genes in OA,” namely PLIN2, PTGS2, VEGFA, and IL6, as shown in Fig. 2C.

### GO and KEGG enrichment analysis results

**Fig. 3:**
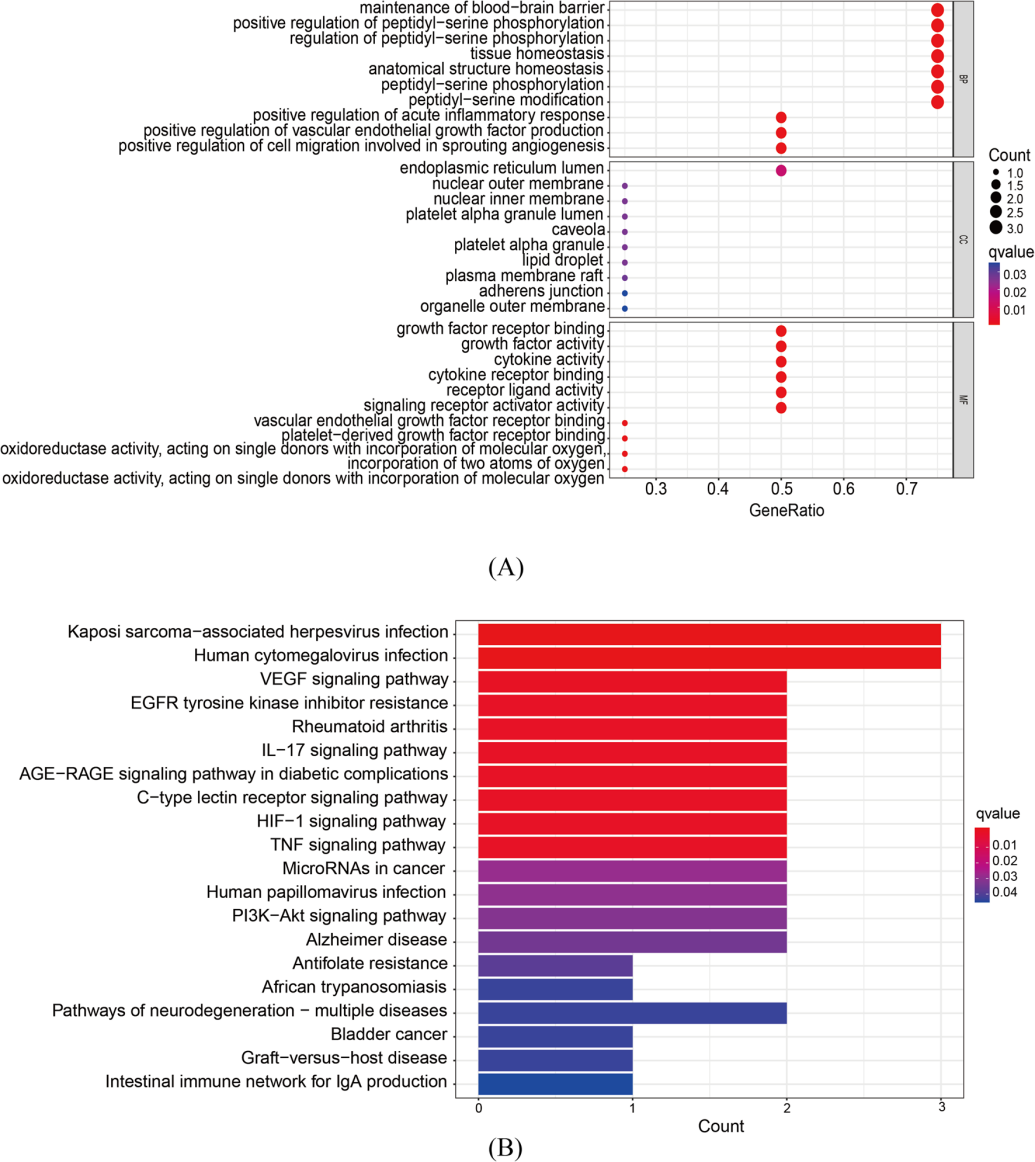
GO enrichment and KEGG enrichment analysis results. **A** Graph of GO enrichment analysis results; the larger the circle in the figure, the larger the number of relevant targets; **B** graph of KEGG enrichment analysis results; the longer the column in the figure, the more the number of related targets; a redder color represents a higher degree of enrichment

GO enrichment results showed that the biological processes were mainly involved in the maintenance of blood-brain barrier, serine phosphorylation, acute tissue homeostasis, and positive regulation of inflammatory response. Cellular components showed that they were mainly present in the inner lumen of endoplasmic reticulum, nuclear outer membrane, nuclear inner membrane and platelet α granule lumen. In terms of molecular function, it plays a role in growth factor receptor activity, cytokine receptor activity, vascular endothelial growth factor receptor binding, etc., as shown in Fig. 3A.

KEGG enrichment analysis showed that puerarin ferroptosis-osteoarthritis common genes were mainly involved in VEGF signaling pathway, IL-17 signaling pathway, C-type lectin receptor signaling pathway, HIF-1 signaling pathway, and TNF signaling pathway. It also plays a role in AGE-RAGE signaling in diabetic complications and in EGFR tyrosine kinase inhibitor resistance, rheumatoid arthritis, Kaposi’s sarcoma-associated herpesvirus infection, and human cytomegalovirus infection, as shown in Fig. 3B.

### Molecular docking results

**Fig. 4:**
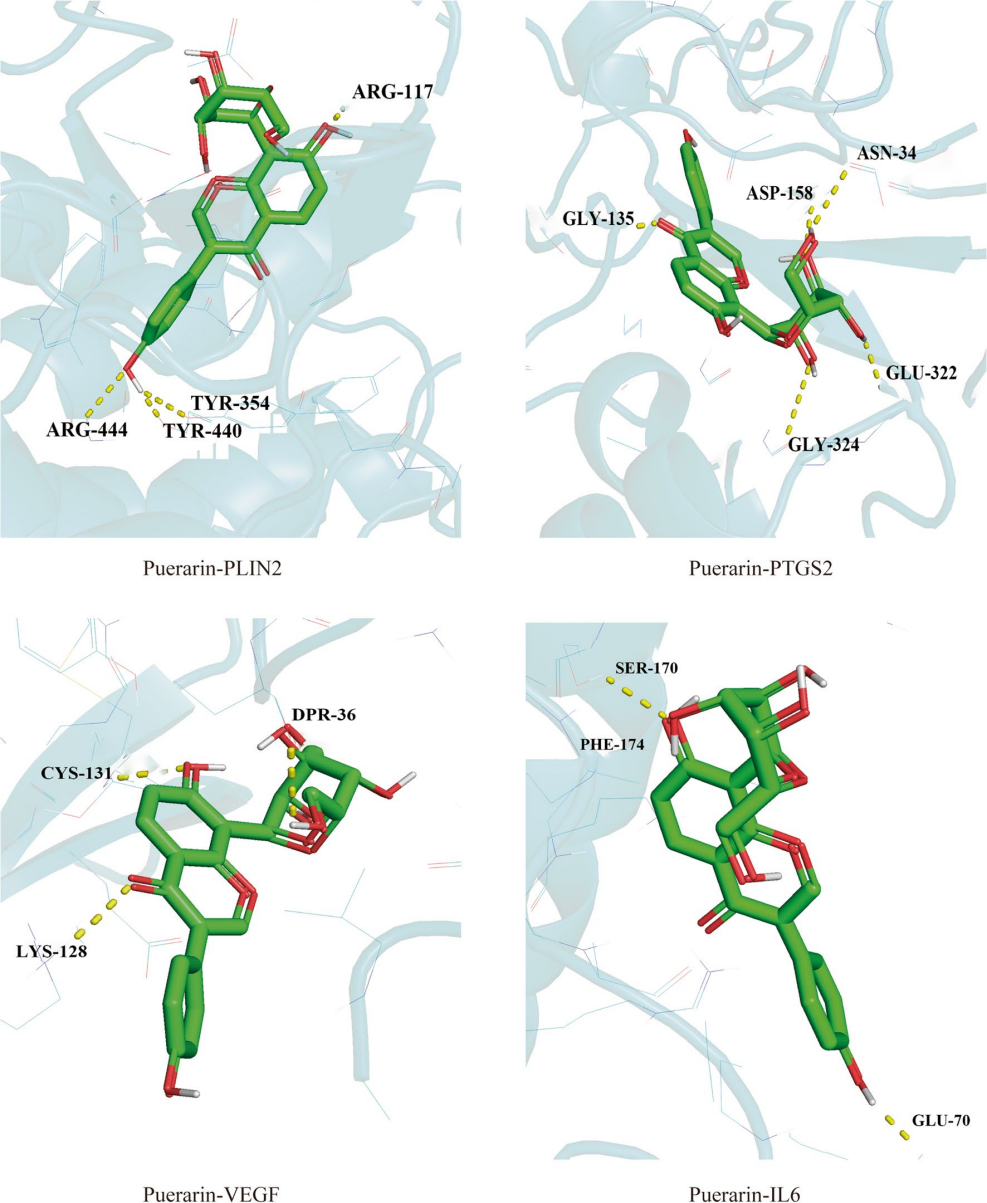
Visualization of docking between puerarin and core target molecules (binding energy ≤−6.9 kcal/mol)

The common genes obtained in “GO and KEGG enrichment analysis results” were used for molecular docking with puerarin, and the results showed that PLIN2, PTGS2, VEGFA, and IL6 all had good affinity with puerarin. The docking model with the lowest binding free energy is regarded as the best binding model, as shown in Fig. 4, and the specific binding free energy values are detailed in Table 1.

**Table 1:** Molecular docking binding free energy values

Project	Gene name	Binding free energy (kcal/mol)
Puerarin	PLIN2	−8.3
	PTGS2	−8.4
	VEGF	−7.7
	IL6	−6.9

### Anti-inflammatory effects of puerarin

Human articular chondrocytes were treated with different concentrations of puerarin (5 μM, 10 μM, 20 μM, 50 μM, and 100 μM) and celecoxib (5 μM, 10 μM, 20 μM, 100 μM, and 200 μM) for 24 h and 48 h, respectively. The results showed that puerarin and celecoxib at the concentration of 10 μM had the highest survival rate of human articular chondrocytes.

**Fig. 5:**
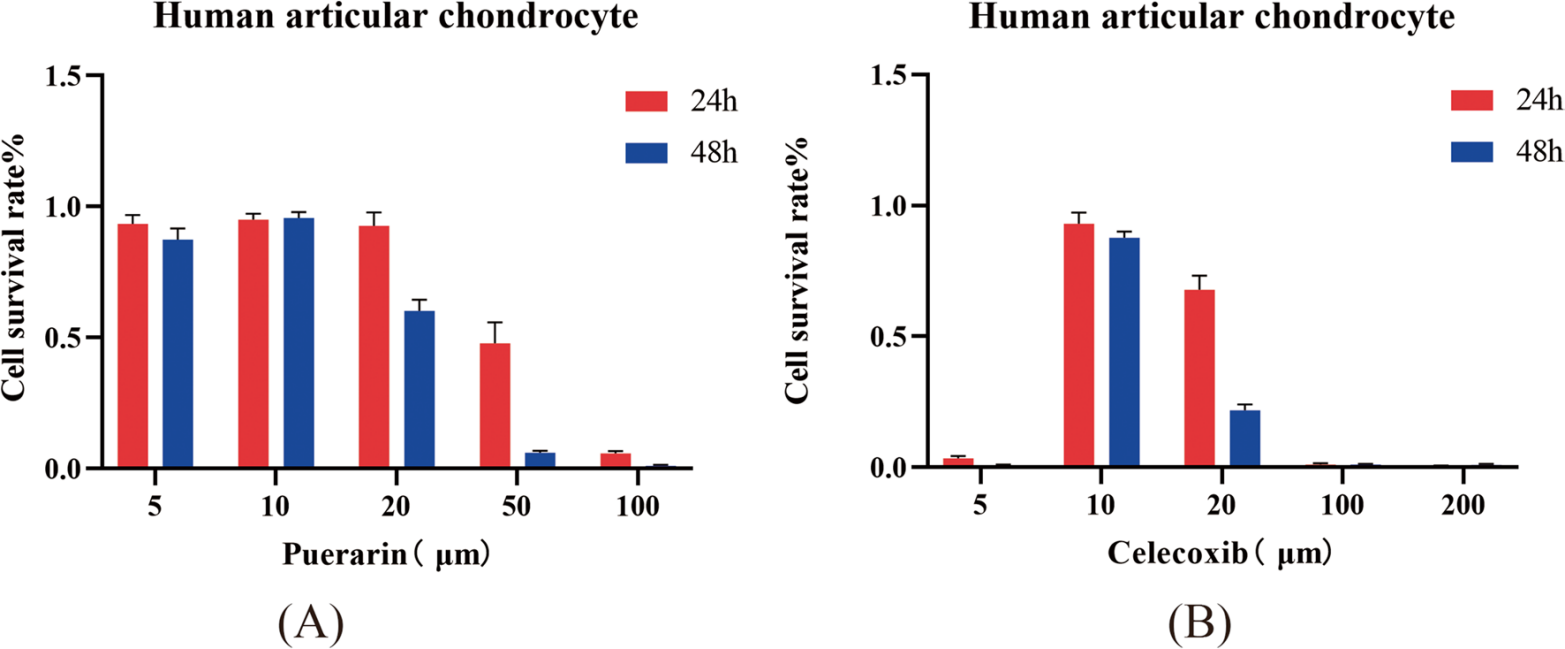
Effect of different concentrations of puerarin **A** and celecoxib **B** on cell viability of human articular chondrocytes damaged by IL-1β

This indicates that puerarin and celecoxib have protective effects against IL-1β-induced injury of human articular chondrocytes, and 10 μM concentration has the best effect, as shown in Fig. 5A and B.

Compared with the control group, the concentrations of IL-1β, IL-6, and TNF-α in the model group were increased (*p* < 0.05). Compared with the model group, the concentrations of IL-1β, IL-6, and TNF-α in chondrocytes were significantly decreased in the celecoxib and puerarin groups (*p* < 0.05). Compared with the celecoxib group, the concentrations of IL-1β and IL-6 were significantly decreased in the puerarin group (*p* < 0.05). The results showed that puerarin could reduce the secretion of related inflammatory factors in human articular chondrocytes and thus play an anti-inflammatory effect, as shown in Table 2.

**Table 2:** Effects of celecoxib and puerarin on IL-1β, IL-6, and TNF-α levels of human articular chondrocytes induced by IL-1β (±s, *n*=10)

Group	IL-1β (pg/mL)	IL-6 (pg/mL)	TNF-α (pg/mL)
Control group	23.06±3.38	7.43±1.80	17.80±3.78
Model group	58.46±6.96^*^	35.16±2.16^*^	58.05±2.80^*^
Celecoxib group	48.01±4.68^#^	25.59±2.98^#^	39.70±4.49^#^
Puerarin group	40.51±7.15^#&^	18.07±1.86^#&^	38.66±5.12^#^

Compared with the control group, *p < 0.05; compared with the model group, ^#^*p* < 0.05; compared with puerarin group, ^&^*p*<0.05

## Discussion

Inflammatory cytokines are crucial for the occurrence and development of OA. The dynamic imbalance between pro-inflammatory cytokines and anti-inflammatory cytokines can lead to abnormal metabolism of knee cartilage and eventually destroy the normal structure of knee joint (Liu et al. 2022). Ferroptosis is closely related to inflammation, which can lead to a significant increase in pro-inflammatory cytokines (TNFα, IL-1β, and IL-6) (Qi et al. 2020), and IL-1β can induce inflammatory damage and ferroptosis of chondrocytes (Wang et al. 2022). Iron accumulation and oxidative stress are common pathological features of OA. Iron homeostasis and lipid peroxidation are closely related to the pathogenesis of OA (Zhang et al. 2022). It is recorded in Shennong Bencao Jing (Shennong’s Classic of Materia Medica) that pueraria treats all bi syndrome, and related prescriptions include Gegen decoction, Gegen Jianghuang San (Liang 2020), and Gegen Jiaguizhi Tang. Puerarin is the main active component of pueraria, which can inhibit iron cell apoptosis and inflammation by reducing lipid peroxidation, downregulating the levels of pro-inflammatory factors (TNF-α, IL-1β, and IL-6), and regulating the levels of iron cell apoptosis–related proteins (Xu et al. 2021). However, the mechanism between puerarin, ferroptosis, and osteoarthritis is still unclear. Exploring the anti-inflammatory effect of puerarin from the perspective of ferroptosis may provide a new and effective treatment for osteoarthritis.

Common targets of puerarin, ferroptosis, and osteoarthritis include PLIN2, PTGS2, VEGF, and IL6. Studies have found that puerarin can improve lipid deposition in the liver of mice by reducing the expression of PLIN2 (Zhou et al. 2022). Lipid metabolic pathways can produce pro-inflammatory substances that are critical for the development and management of osteoarthritis (OA). PTGS2, also known as COX-2, is closely related to various inflammatory responses of the body, and studies have shown that puerarin can inhibit its expression (Hu et al. 2011; Liu et al. 2018). VEGF is a mediator of angiogenesis and inflammation, and angiogenesis and inflammation are closely related under many physiological and pathological conditions (Shaik-Dasthagirisaheb et al. 2013). IL-6 is an inflammatory cytokine, and its level will increase under most inflammatory conditions. IL-6 is an inflammatory cytokine, and its level is increased in most inflammatory states (Rose-John 2022). Most of these common target proteins were involved in inflammatory response, which verified the correlation between the occurrence and development of OA and inflammatory response. Therefore, it can be speculated that puerarin may regulate ferroptosis and interfere with the development of OA by acting on its targets.

GO analysis showed that puerarin treatment of OA was mainly related to biological processes, including serine phosphorylation, acute tissue homeostasis, and positive regulation of inflammatory response. Receptor-interacting serine/threonine protein kinase 1 (RIPK1) is a cytosolic protein kinase that regulates a variety of inflammatory and cell death pathways (Tu et al. 2022). Inflammation is a defense response of the body to stimulation, which plays a key role in restoring tissue homeostasis. Some scholars have proposed the hypothesis of enhancing homeostatic mechanisms to maintain joint health and reduce the risk of arthritis (Hao and Zhang 2015). Therefore, based on ferroptosis, puerarin intervention in OA is related to the above biological processes such as inflammatory response.

KEGG results showed that VEGF signaling pathway, HIF-1 signaling pathway, IL-17 signaling pathway, and TNF signaling pathway were mainly involved in the treatment of OA by puerarin. Studies have found that the expression of VEGF and HIF-1α protein in articular chondrocytes of OA patients is significantly increased, suggesting that upregulation of the target gene VEGF expression is a possible regulatory mechanism of HIF-1α in the pathogenesis of OA (Xiao-yan et al. 2010). Studies have shown that the abnormal expression of IL-17 is closely related to the pathogenesis and pain degree of OA, and blocking the IL-17 signal transduction pathway may relieve the pain related to knee osteoarthritis (Liu et al. 2015). TNF is a pro-inflammatory cytokine, which can activate inflammatory cytokines such as IL-1β, IL-6, and TNF-α, and regulate local inflammatory response and cartilage tissue remodeling. In this study, TNF signaling pathway was found to be significantly expressed, suggesting that puerarin could not only regulate the inflammatory response, but also inhibit the degradation of articular cartilage and play a therapeutic role in OA.

Therefore, based on ferroptosis, puerarin is involved in multiple signaling pathways to inhibit inflammatory response and exert anti-OA effects.

In this study, chondrocytes were induced with IL-1β to mimic osteoarthritis in vitro and treated with different doses of puerarin and celecoxib. The results showed that at the concentration of 10 μM, puerarin and celecoxib had the best protective effect on IL-1β-induced human articular chondrocytes injury. Both celecoxib and puerarin could reduce the concentrations of IL-1β, IL-6, and TNF-α in chondrocytes, indicating that puerarin could inhibit IL-1β-induced inflammatory response of chondrocytes by reducing inflammatory factors.

In conclusion, puerarin can improve the inflammatory response of chondrocytes and combine with ferroptosis mechanism, and its targets may be related to PLIN2, PTGS2, VEGF, and IL6. Molecular docking suggested that puerarin had a good binding effect with the core targets. It plays a synergistic role through biological processes and signaling pathways such as VEGF signaling pathway, HIF-1 signaling pathway, IL-17 signaling pathway, and TNF signaling pathway, serine phosphorylation, acute tissue homeostasis, and positive regulation of inflammatory response. The relevant mechanism needs to be further verified by in vitro and in vivo experiments.

## Data Availability

All data supporting the article is provided in this article.
